# Social Support Mediates the Relationship between Body Image Distress and Depressive Symptoms in Prostate Cancer Patients

**DOI:** 10.3390/ijerph19084825

**Published:** 2022-04-15

**Authors:** Cristiano Scandurra, Benedetta Muzii, Roberto La Rocca, Francesco Di Bello, Mario Bottone, Gianluigi Califano, Nicola Longo, Nelson Mauro Maldonato, Francesco Mangiapia

**Affiliations:** 1 Department of Neuroscience, Reproductive Sciences and Dentistry, University of Naples Federico II, 80131 Naples, Italy; roberto.larocca@unina.it (R.L.R.); fran.dibello12@gmail.com (F.D.B.); bottone.mario@fastwebnet.it (M.B.); gianl.califano2@gmail.com (G.C.); nicola.longo@unina.it (N.L.); nelsonmauro.maldonato@unina.it (N.M.M.); mangiapiaf@gmail.com (F.M.); 2 Department of Humanistic Studies, University of Naples Federico II, 80133 Naples, Italy; benedetta.muzii@unina.it

**Keywords:** prostate cancer, social support, body image, depressive symptoms, mediation

## Abstract

Treatments for prostate cancer (PCa), the second most common cancer in men, may affect the body image (BI) of patients, increasing the risk of negative mental health outcomes. However, an enabling social support network may be a protective factor against the effects of BI distress on health. Therefore, the present study examined the mediating role of social support in the relationship between BI distress and depressive symptoms. Data were retrospectively collected from 197 PCa patients aged from 48 to 79 years (*M* = 67.19; *SD* = 6.83). The statistical package for the social sciences with PROCESS Macro was used to assess the direct and mediating effects with bias-corrected bootstrapping (10,000 samples). Results showed that BI distress was positively associated with depressive symptoms and that social support partially mediated this relationship. Moreover, among the different sources of social support, only friend support significantly mediated the association between BI distress and depressive symptoms. This study sheds light on the crucial role of social support as a dimension that can promote health in PCa patients.

## 1. Introduction

Prostate cancer (PCa) is the second most common cancer in men and accounted for one in five new diagnoses in 2020 [1]. Treatment protocols for PCa consist of surgery, chemotherapy, hormone therapy, radiation, or a combination thereof, but can involve life-altering physical side effects such as urinary incontinence, energy levels, and erectile dysfunction. It is well known that physical stress can affect mental health and well-being. This is one of the main reasons why PCa survivors have a significantly higher likelihood of screening positive for depressive symptoms [2] as well as a higher risk of death compared to patients without depression [3] and higher suicide mortality when compared to the general population [4].

One of the psychological dimensions most affected in cancer patients is body image (BI). BI is a complex dimension that includes perceptions, feelings, thoughts, and attitudes toward one’s body and its functioning [5]. For PCa patients, treatments can result in genital shrinkage (e.g., loss of penile length and/or testicular mass) and weight changes [6], and these conditions can negatively impact BI, including feelings and appraisals toward the body, as well as physical strength and vitality. Previous studies have shown an association between BI distress and mental health [7]. Specifically, PCa patients suffering from BI distress are more likely to report a poor quality of life [8] and worse mental health [9,10,11].

However, there is a large body of research on cancer health that demonstrates that social support can be a protective factor against the effects of BI distress on health. Social support can be defined as the quality and function of social relationships such as the actual support received or the perceived availability of help [12]. There are several types of social support, including emotional (e.g., the provision of empathy, care, and love by family and close friends), informational (e.g., advice from members of a social network who can help the patient respond to personal needs), and instrumental (help in the form of time, money, or practical support) [13,14]. Social support plays a crucial role in improving the quality of life and well-being of cancer patients [15]. Regarding the relationship between social support and BI, according to the Acceptance Model [16], acceptance of the body by others can protect people from adopting a self-objectifying attitude, which contributes to people having positive feelings regarding their bodies. Thus, according to the Stress-Buffering Model [17,18], social support may have a kind of “buffer effect” on cancer patients’ acceptance of their own BI [19], especially for patients undergoing invasive surgery [20]. Specifically, the perception of having significant sources of social support (e.g., from family, friends, or significant others) has been found to be negatively associated with BI distress as these sources can help people cope with BI concerns thanks to the feeling of being recognized and mirrored by others and not being alone [21,22,23,24].

On the other hand, recent research has begun to consider social support as a potential mediator, rather than a moderator, between physical impairments or stressful psychological experiences and health outcomes [25,26,27]. Indeed, as suggested by Paterson et al. [28], understanding the mechanisms by which social support affects health and quality of life in PCa patients is critical to developing appropriate theory-based interventions that effectively improve health in this population. Although there are no solid theories yet about the mediating role of social support [28], previous research has shown that perceived social support is strongly associated with feelings of belonging, self-efficacy, and emotional stability [18,29], which are general psychological processes (i.e., mediators) explaining why some individuals are at higher risk of developing negative mental health outcomes [30]. For example, previous research has found that people without cancer who have high levels of stress are more likely to have lower social support than their counterparts due to their lower levels of satisfaction with themselves, which has a negative impact on their health [31,32]. In this regard, cancer can lead to feelings of self-embarrassment and shame, which can be a barrier to disclosure. To this end, Ettridge et al. [33] highlighted that PCa patients do not seek social support due to expected awkwardness, unwanted compassion, and BI distress. Moreover, these factors may weaken patients’ significant relationships and consequently promote isolation, which increases the risk of depression [34]. Along these lines, in a sample of patients with skin tumors, Pereira et al. [35] found that social support mediates the relationship between family stress and psychological morbidity and quality of life, while in a sample of breast cancer patients, Hsu et al. [36] found that social support mediates the relationship between BI distress and resilience, a dynamic process closely associated with health and well-being.

**Figure 1:**
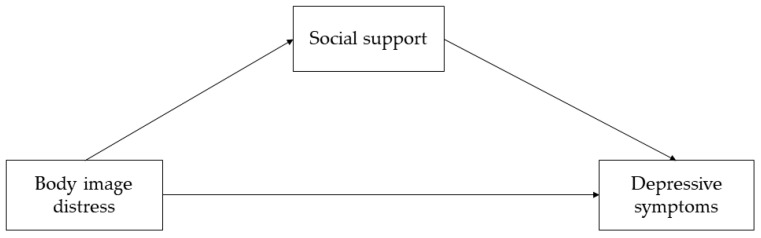
The hypothesized mediation model.

Notwithstanding these theoretical premises, no previous studies have examined the mediating role of social support in the relationship between BI distress and depressive symptoms in PCa patients. Therefore, the aim of the current study was to examine a series of hypotheses that combined BI distress, social support, and depressive symptoms in a mediation model (Figure 1). Specifically, we hypothesized that: (1) BI distress is positively associated with depressive symptoms; (2) social support is negatively associated with depressive symptoms; and (3) social support mediates the relationship between BI distress and depressive symptoms.

## 2. Materials and Methods

### 2.1. Participants and Procedures

Data were retrospectively collected from 197 PCa patients recruited at the Urology Department of the University Hospital of Naples Federico II. Patients with a histologically confirmed PCa diagnosis and who had undergone radical prostatectomy or radiotherapy for clinically localized PCa between 2013 and 2021 were contacted and asked to participate in an online survey.

All participants provided their consent to participate by clicking “I agree to participate in the study”. All questions were mandatory to avoid missing data. However, participants were informed of their right to withdraw from the survey at any time and for any reason.

The study was designed in respect of the principles of the Declaration of Helsinki on Ethical Principles for Medical Research Involving Human Subjects and was approved by the ethical committee of the School of Medicine and Surgery of the University of Naples Federico II (protocol number 261/2019).

### 2.2. Measures

#### 2.2.1. Demographics and Clinical Characteristics

We collected information on age, ethnicity (Caucasian vs. non-Caucasian), education level (≤high school vs. ≥university), sexual orientation, and marital status (with partner vs. without partner). To assess PCa severity, we also collected information on primary treatment (surgery only vs. radiotherapy only vs. surgery combined with androgen deprivation therapy vs. radiotherapy combined with surgery), serum level of prostate-specific antigen (PSA) (ng/mL), Gleason score (from 6 to 10), and ISUP (International Society of Urological Pathology) grade (from 1 to 5) [37].

#### 2.2.2. Body Image Distress

The BI distress was assessed using the *Body Image Scale* (BIS) [38], a 10-item cancer-specific scale that assesses perceptions of one’s appearance by examining affective (e.g., feeling less masculine, feeling less attractive), behavioral (e.g., finding it difficult to look in the mirror), and cognitive (e.g., satisfaction with appearance) dimensions of distress. The original scale included an item on dissatisfaction related to the surgical scar. As suggested by Langelier et al. [39], we deleted this item because this experience is not universal among PCa patients. Response options range from 0 (not at all) to 3 (very much), with higher scores indicating greater BI distress. The *α* coefficient in the current study was 0.88.

#### 2.2.3. Depressive Symptoms

Depressive symptoms were assessed using the *Patient Health Questionnaire Depression Scale-9* (PHQ-9) [40], a 9-item scale measuring the frequency and severity of depressive symptoms in the previous 2 weeks. Response options range from 0 (not at all) to 3 (nearly every day), with higher scores indicating greater severity and a cut-off score of 10 suggesting major depressive disorder. The *α* coefficient in the current study was 0.84.

#### 2.2.4. Social Support

Social support was assessed using the *Multidimensional Scale of Perceived Social Support* (MSPSS) [41], a 12-item scale that measures the extent of perceived support from family, friends, and significant others. Response options range from 1 (very strongly disagree) to 7 (very strongly agree), with higher scores reflecting greater perceived social support. The *α* coefficients were 0.92 for the significant others subscale and 0.93 for all other subscales, including the total scale.

### 2.3. Statistical Analyses

Statistical analyses were performed using the Statistical Package for the Social Sciences (SPSS 27).

First, participants’ clinical characteristics, descriptive statistics, and bivariate correlations between BI distress, social support, and depressive symptoms were calculated. Then, a mediation model analysis was conducted to test the direct and mediating effects of BI distress and social support on depressive symptoms. In this model, age, marital status, time of intervention, and disease severity indices (PSA, Gleason score, and ISUP grade) were included as control variables.

The statistical package for the social sciences (SPSS) with PROCESS Macro (i.e., Model 4) [42] was used to assess the statistical significance of the direct and mediating effects with bias-corrected bootstrapping (10,000 samples) and 95% confidence intervals (*CI*). According to Hayes [42], the indirect effect can be considered significant if the upper and lower boundaries of the bias-corrected 95%*CI* do not contain zero. Before performing the analyses, the continuous variables were centered.

## 3. Results

### 3.1. Participants’ Characteristics

As shown in Table 1, patients ranged in age from 48 to 79 years (*M* = 67.19; *SD* = 6.83). Furthermore, most patients had an educational level ≤ high school (*n* = 148; 75.1%) and were Caucasian (*n* = 193; 98%), heterosexual (*n* = 191; 96.9%), and in a stable relationship (*n* = 167; 84.8%). Regarding clinical characteristics, the time of PCa treatment ranged from a few months to eight years (*M* = 1.58 years; *SD* = 1.47).

The ISUP grades and Gleason scores were as follows: (a) ISUP grade 1 and Gleason score 6 (*n* = 22; 11.2%); (b) ISUP grade 2 and Gleason score 7 (3 + 4) (*n* = 33; 16.8%); (c) ISUP grade 4 and Gleason score 8 (*n* = 86; 43.6%); (d) ISUP grade 5 (*n* = 24; 12.2%), including 21 (10.7%) with Gleason score 9 and three (1.5%) with Gleason score 10. The median PSA level was 7.49 ng/mL. In addition, 15% (*n* = 30) of participants met the clinical cutoff for possible major depressive disorder.

### 3.2. Descriptive Statistics and Bivariate Correlations

Means, standard deviations, ranges, and bivariate correlations between BI distress, social support, and depressive symptoms are shown in Table 2. The results showed that all variables were correlated with each other. Specifically, BI distress correlated negatively with social support and positively with depressive symptoms, whereas social support correlated negatively with depressive symptoms.

### 3.3. Direct and Indirect Effects of Body Image Distress and Social Support on Depressive Symptoms

In support of our first hypothesis, we found that BI distress was negatively associated with social support (*β* = −0.08, standard error (*SE*) = 0.01, 95%*CI* (−0.09, −0.05), *p* < 0.001) and positively associated with depressive symptoms (*β* = 0.33, *SE* = 0.05, 95%*CI* (0.22, 0.44), *p* < 0.001).

In support of our second hypothesis, the results showed that social support was negatively associated with depressive symptoms (*β* = −1.27, *SE* = 0.32, 95%*CI* (−1.91, −0.63), *p* < 0.001).

**Figure 2:**
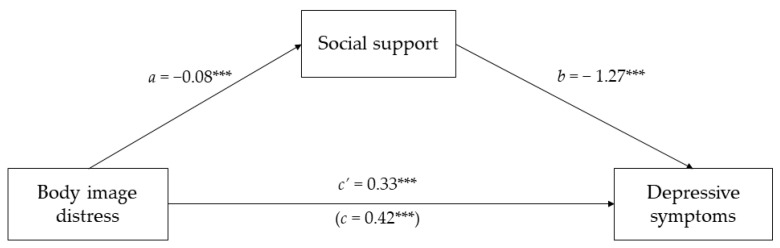
The mediated effect of social support on the relationship between body image distress and depressive symptoms. *** *p* < 0.001. All values are beta coefficients.

When we included social support as a mediator, there was a significant overall effect (*β* = 0.42, *SE* = 0.05, 95%CI (0.31, 0.52), *p* < 0.001), while the direct effect remained significant, indicating a case of partial mediation and confirming our third hypothesis. Indeed, the indirect effects showed that social support significantly mediated the association between BI distress and depressive symptoms (*β* = 0.09, *SE* = 0.03, 95%*CI* (0.04, 0.15)). In addition, BI distress and social support explained a significant proportion of the variance in depressive symptoms (*F* (7, 184) = 1.04, *p* < 0.001, *R*^2^ = 0.27). The results of the mediation model are shown in Figure 2.

None of the control variables showed a significant association with depressive symptoms; age (*β* = −0.04, *SE* = 0.05, *p* = 0.45), time of intervention (*β* = 0.10, *SE* = 0.23, *p* = 0.65), ISUP grade (*β* = 0.21, *SE* = 1.16, *p* = 0.86), PSA (*β* = −0.07, *SE* = 0.03, *p* = 0.07), Gleason score (*β* = −0.34, *SE* = 1.49, *p* = 0.81), and stable partner (*β* = −0.60, *SE* = 0.87, *p* = 0.49).

Finally, because the MSPSS consists of multiple sources of social support, we examined whether all dimensions of social support mediated the relationship between BI distress and depressive symptoms. Therefore, we created a new model using the three subscales of the MSPSS as mediators. Only significant indirect effects are reported for parsimony. In contrast to perceived support from family (*β* = 0.03, *SE* = 0.04, 95%*CI* (−0.06, 0.89)) and significant others (*β* = 0.01, *SE* = 0.04, 95%*CI* (−0.08, 0.10)), we found that only perceived support from friends significantly mediated the relationship between BI distress and depressive symptoms (*β* = 0.09, *SE* = 0.04, 95%*CI* (0.03, 0.17)).

## 4. Discussion

The current study aimed to investigate the mediating role of social support in the relationship between BI distress and depressive symptoms in a group of PCa patients. The results confirmed our hypotheses. To our knowledge, this is the first study that tested this mediation model in this specific population (i.e., PCa patients), highlighting potentially significant pathways through which BI discomfort could influence depressive symptoms in men with PCa.

In support of our first hypothesis, we found that BI distress was negatively associated with depressive symptoms. This finding is consistent with previous studies that have found a significant association between these dimensions in cancer patients [9,43,44,45,46]. With regard to PCa patients, in a qualitative meta-synthesis on BI, self-esteem, and sense of masculinity, Bowie et al. [7] highlighted that BI is particularly influenced by two factors, i.e., the changes in function that these patients experience as a result of diagnosis and treatment and the changes in how others treat their bodies. Regarding the first factor, Bowie et al. [7] pointed out that many PCa patients perceive their body as a source of shame after the interventions [47] and that the body is perceived as incomplete due to the removal of the prostate or sexual impairment [48,49]. Instead, regarding the second factor, Bowie et al. [7] highlighted that being touched and handled by physicians can cause feelings of vulnerability and shame in PCa patients [50,51,52] who may furthermore perceive their bodies as old due to the premature aging that the treatments cause [53]. Thus, all the above subjective experiences may represent a serious risk factor for the development of depressive symptoms in PCa patients.

As for the second hypothesis, we found that social support was negatively associated with depressive symptoms. This finding is consistent with a well-established research tradition highlighting that high perceived social support is consistently associated with better mental health outcomes due to the perceived availability of friends, family, and significant others as sources that provide material and psychological support in times of need [54,55]. This finding has also been widely confirmed in cancer research, underscoring that social support has the potential to reduce cancer-related stress and psychological problems [56,57,58]. Similar results were found in PCa patients, in whom a stronger social support network can improve mental health by facilitating the use of adaptive coping strategies [59], which confirms that social support is an important protective factor for PCa patients.

Finally, regarding the third hypothesis, we found that social support—particularly support from friends—partially mediated the relationship between BI distress and depressive symptoms. This is the most innovative finding of the current study, which can be interpreted with the help of recent research that considers social support as a mediator between stressful experiences, such as BI distress, and health [24,31,32,33,34,35,36]. This finding seems to indicate that PCa patients with low BI distress levels can maintain higher social support than their counterparts and that this would reduce the likelihood of developing depressive symptoms. Thus, it could mean that alleviating BI distress in PCa patients may encourage them to activate their social support networks, which in turn would lead to better mental health. This finding may provide valuable guidance for the implementation of psychological interventions to improve the mental health of PCa patients. Indeed, avoiding or reducing BI distress could be a preventive therapy to help these individuals improve their health. In addition, getting PCa patients to activate their own resources to take advantage of the existing social support network could be effective in reducing the risk of developing negative mental health outcomes.

Interestingly, what has been said so far about social support seems to only apply to perceived support from friends. Consistent with evidence that friendship is a powerful source of healing from physical and mental illness in people with cancer [60], it is plausible to hypothesize that PCa patients feel more comfortable talking about their bodies with friends than with family members. This finding is consistent with Cicero et al. [61] who found that support from friends promoted an adaptation to cancer more than support from family. This could be due to the fact that family members are affected by the stressors associated with the disease while friends are relieved of the burden of care and can trigger strategies in the wider network that lead to adaptation to the disease. Another possible explanation is that this finding has to do with masculinity, which is a very significant dimension in PCa, as patients often report that the treatment-related physical changes have affected their masculine self-esteem [62,63]. Presumably, and given the particular context in which this study was conducted (i.e., Italy), it seems plausible that it might be more reassuring for a man to talk about this discomfort with a same-sex friend than with a partner, for example. However, although Italy is a rather conservative country where people show high levels of heteronormative attitudes [64,65,66,67], especially among the older generations such as the participants in the current study whose average age is almost 70, our interpretative hypothesis of the phenomenon needs to be empirically deepened as it is likely subject to gender and sexual biases.

The results of the current study should be read while considering significant limitations. First, because of the cross-sectional design, it was not possible to draw firm conclusions about the causality and direction of relationships among variables. Longitudinal studies would be needed to overcome this limitation and to determine whether social support mediates the relationship between BI distress and depressive symptoms over the course of illness. Second, almost all participants were Caucasian and heterosexual; therefore, it was not possible to include these identity dimensions as control variables in the tested model. Third, because of the very small number of participants who received combined treatment (e.g., surgery and radiotherapy) or radiotherapy alone, we were unable to control our results for the type of treatment. Future studies should replicate this study and expand the sample in terms of both sociodemographic characteristics and clinical features. Finally, of the different types of social support, we studied only perceived support and did not consider objective support or satisfaction with the support received. Future studies should use more composite measures of social support and analyze its different forms.

## 5. Conclusions

This study extended the findings on the complex interactions between BI distress, social support, and mental health in PCa patients. Overall, our study sheds light on the crucial role of social support as a dimension that can promote health in PCa patients.

## Figures and Tables

**Table 1:** Socio-demographic and clinical characteristics of PCa patients (*n* = 197).

Variable	Patients *n* (%) or *M* ± *SD*
*Age*	67.19 ± 6.83
Range	48–79
*Education*	
≤high school	148 (75.1)
≥university	49 (24.9)
*Ethnicity*	
Caucasian	193 (08)
Non-Caucasian	4 (2)
*Stable relationship*	
Yes	167 (84.8)
No	30 (15.2)
*Sexual orientation*	
Heterosexual	191 (96.9)
Non-heterosexual	7 (3.1)
*Time of treatment* (in years)	1.58 ± 1.47
*Type of intervention*	
Surgery only	188 (95.4)
Surgery in combination with ADT	7 (3.6)
Radiation therapy only	2 (1)
Radiation therapy in combination with surgery	2 (1)
*ISUP grade/Gleason score*	
ISUP grade 1/Gleason score 6	22 (11.2)
ISUP grade 2/Gleason score 7 (3 + 4)	33 (16.8)
ISUP grade 4/Gleason score 8	86 (43.6)
ISUP grade 5/Gleason score 9	21 (10.7)
ISUP grade 5/Gleason score 10	3 (1.5)
*PSA* (median and range)	7.49 (1−103)

Notes: *M* = mean; *SD* = standard deviation; PCa = prostate cancer; ADT = androgen deprivation therapy; ISUP = International Society of Urological Pathology; *PSA* = prostate-specific antigen.

**Table 2:** Descriptive statistics and bivariate correlations between body image distress, social support, and depressive symptoms.

	1	2	3	*M* ± *SD*	Ranges
1. Body image distress	-			7.88 ± 6.32	0−27
2. Social support	−0.37 ***	-		5.64 ± 1.08	1−7
3. Depressive symptoms	0.52 ***	−0.38 ***	-	5.41 ± 5.01	0−24

Note: *M* = mean; *SD* = standard deviation. *** *p* < 0.001.

## Data Availability

Anonymized data will be made available upon reasonable request to the corresponding author.
